# Vestibular dysfunction is an important contributor to the aging of visuospatial ability in older adults–Data from a computerized test system

**DOI:** 10.3389/fneur.2022.1049806

**Published:** 2022-11-17

**Authors:** Xuehao Zhang, Yan Huang, Yuqi Xia, Xiaotong Yang, Yanmei Zhang, Chaogang Wei, Hang Ying, Yuhe Liu

**Affiliations:** ^1^School of Medical Technology and Information Engineering, Zhejiang Chinese Medical University, Hangzhou, China; ^2^Department of Otolaryngology, Head and Neck Surgery, Peking University First Hospital, Beijing, China; ^3^Department of Otolaryngology, Head and Neck Surgery, Beijing Friendship Hospital, Capital Medical University, Beijing, China

**Keywords:** vestibular dysfunction, recurrent vertigo, aging, visuospatial ability, computerized test system

## Abstract

**Background:**

A convergence of research supports a key role of the vestibular system in visuospatial ability. However, visuospatial ability may decline with age. This work aims to elucidate the important contribution of vestibular function to visuospatial ability in old adults through a computerized test system.

**Methods:**

Patients with a clinical history of recurrent vertigo and at least failed one vestibular test were included in this cross-sectional study. Healthy controls of three age groups: older, middle-aged, and young adults were also involved. Visuospatial cognitive outcomes including spatial memory, spatial navigation, and mental rotation of all the groups were recorded. Comparing the performance of the visuospatial abilities between patients and age-matched controls as well as within the controls.

**Results:**

A total of 158 individuals were enrolled. Results showed that patients performed worse than the age-matched controls, with the differences in the forward span (*p* < 0.001), the time of the maze 8 × 8 (*p* = 0.009), and the time of the maze 12 × 12 (*p* = 0.032) being significant. For the differences in visuospatial cognitive outcomes within the controls, the younger group had a significantly better performance than the other groups. The older group and the middle-aged group had comparable performances during all the tests.

**Conclusions:**

Older patients with vestibular dysfunction had more difficulties during visuospatial tasks than age-matched controls, especially in spatial memory and spatial navigation. Within the controls, younger adults did much better than other age groups, while older adults behaved similarly to middle-aged adults. It is a valuable attempt to computerize the administration of tests for visuospatial ability.

## Introduction

A growing body of research suggests that the vestibular system is involved in both reflexes at the brainstem level and in complex cognitive processes (1, 2). This seems plausible since, compared to other sensory information, the perception of vestibular input is more dependent on the individual's own continuous and dynamic mental activity (3). Anatomical evidence indicates that vestibular information is further transmitted to the cerebral cortex *via* the vestibular nucleus to form complex, conscious mental representations, enabling individuals to perceive their position and state in the real-time environment and to complete real-life orientation and navigation (4–7).

Researchers have carried out valuable explorations in vestibular-related cognitive domains. Wherein, visuospatial ability is more closely related to the vestibular system. It refers to the ability to understand and organize information about the environment in two- and three-dimensional space, which includes a variety of skills such as spatial memory, spatial navigation, and mental rotation (8). A study has found that compared to controls, patients with bilateral vestibulopathy (BVP) show poorer spatial learning ability and more severe spatial anxiety in the virtual Morris Water Task (VMWT)—a computerized version of the Morris Water Maze (MWT), which is used for assessing visuospatial ability of rats (9). Additional studies have shown that BVP patients have difficulty completing mental rotation tasks (10, 11). In addition, vestibular stimuli (e.g., the rotary chair test) have been shown to be able to affect individuals' ability to perform mental rotation tasks (12–14). Some studies have shown that even unilateral vestibular dysfunction may also lead to a decline of visuospatial ability (15–17).

Numerous neuroimaging studies have further established the physiological basis for the connection between the vestibular system and cognition (3, 18). A wider vestibular network in the brain has been identified. Vestibular information forms extensive vestibular-cortical projection areas in the cerebral cortex *via* the thalamus, including frontal regions that are highly relevant to cognitive function (19). A functional near-infrared imaging study found lower activation of the prefrontal cortex in patients with visual vertigo (VV) than in controls during dual-task performance (20). Most of these projection areas play an important role in spatial cognitive tasks. Among them, the hippocampus is considered to be a cardinal structure involved in vestibular-related cognitive functions. The vestibular input related to the environment (e.g., spatial memory, head movements, spatial learning, etc.) is transmitted to the hippocampal-entorhinal cortex through different pathways, then further affects the firing activity of Grid cells, Place cells and HD cells in the hippocampus (21). It was found that the hippocampal volume atrophy seen in patients with bilateral vestibular dysfunction was closely related to spatial memory and spatial navigation deficits (22–24). In addition to the anatomical link, it has been theorized that patients with vestibular dysfunction may need to compensate for visual acuity, balance, and orientation to maintain normal movement, thereby increasing the cognitive load (25).

However, at present, the findings of vestibular-related cognitive abilities are difficult to converge, probably for the following reasons: (1) Existing assessment dimensions are relatively broad, with visuospatial ability accounting for a small proportion, which may be insufficient to reflect the impact of vestibular function; (2) Various types of tools for assessing visuospatial ability make the results of different studies incomparable; (3) Confounding factors, such as hearing status, anxiety and depression, are not strictly controlled, which may also affect the conclusions.

Visuospatial abilities also decline with age. A study found that spatial orientation and navigation abilities decline with age (26). Older populations performed worse than younger populations in the spatial memory task of recalling and replicating visual sequences (27, 28). An interesting study (29), which used a mobile device-based video game program to collect the largest spatial navigation dataset to date, assessed the spatial navigation abilities of a normal population in a virtual environment. The results showed a linear age-related decline in navigation abilities between the ages of 19 and 60. Given the connection between vestibular function, aging, and visuospatial ability, it is necessary to distinguish whether the decline in visuospatial ability regarding older patients with vestibular dysfunction is more due to aging or to vestibular dysfunction.

This study specifically aims to clarify that the vestibular dysfunction is an important factor affecting visuospatial ability in addition to age and whether it is reflected differently in each sub-dimension. Also, to further represent the effect of aging, we included healthy controls of three age groups: older, middle-aged, and young adults. The impact of vestibular function on visuospatial ability was investigated by comparing the performance of patients with age-matched controls and the effect of aging was investigated by comparing the performance within the control group. Moreover, in this study, we developed a computerized Visuospatial Cognition Assessment System (VCAS), which aims to comprehensively assess the visuospatial ability of participants. The mobile terminal presentation of VCAS will give participants a better human-computer interaction experience. It is noted that such computerized assessment has been shown to have similar results to that performed in the real world (30).

## Materials and methods

### Participants

We recruited patients over 60 years old from the outpatient clinic for otolaryngology head and neck surgery at the Peking University First Hospital from December 2021 to May 2022. Patients underwent the appropriate vestibular function tests purely for clinical purposes, including: air-conducted cervical vestibular-evoked myogenic potentials (c-VEMP), video head impulse tests (v-HIT), posturography, videonystagmography (VNG) with bithermal caloric tests. All tests were performed by the same clinical technician. Among them, c-VEMP evaluates saccular function, recording from the sternocleidomastoid (SCM) muscles ipsilateral to the stimulated ear in response to a short pure tone (100 dB SPL) delivered monaurally through insert headphones. Take a 10 dB step until no recognizable P1 and N1 waves (first positive and negative wave with latency ranging from 13 to 23 ms) can be seen. The lower frequencies function of the lateral semi-circular canals was evaluated by v-HIT and bithermal caloric tests. During the v-HIT test, subjects were 1.2 m away from the visual target. Vestibulo-ocular reflex (VOR) gain is defined as the ratio of the angular velocity of eye movement to the angular velocity of head movement. Caloric irrigation was conducted by using cold air at 24°C and hot air at 50°C. Each irrigation was performed after the evoked nystagmus had completely disappeared. Patients who met the following criteria were included in the group of older vertigo patients (OVP).

Inclusion criteria for clinical patients were:

(1) Had a clinical history of recurrent vertigo.

(2) Failed at least one of the following vestibular function tests:

- no recognizable P1 and N1 waves can be seen in either test ear at 100 dB SPL and/or bilateral asymmetry ratio (AR) of amplitude ≥ 1.6, measured by the c-VEMP.
- horizontal angular VOR gain < 0.8 (< 0.7 for vertical direction) with saccade wave, measured by the v-HIT.
- vertigo and characteristic positional nystagmus (torsional nystagmus in the Dix-Hallpike test, horizontal nystagmus in the Roll test) during the posturography.
- reduced caloric response (sum of bithermal, 24 and 50°C maximum peak slow phase velocity (SPV) on each side < 12°/s), and/or unilateral weakness (UW) ≥ 25%.

(3) Subjects might suffer from a post-lingual hearing loss.

To better illustrate the effect of age on visuospatial ability, three types of control participants were recruited, including a young group (YC; 18–44 years old), a middle-aged group (MAC; 45–59 years old), and an older group (OC; 60 years old and above). Some of them were recruited from society, and some were from the examiner's friends and hospital staff. The inclusion criteria for the control group were: (1) 18 years of age and above; (2) had no history of benign paroxysmal positional vertigo (BPPV), meniere disease (MD), vestibular neuritis (VN), vestibular migraine (VM), and other diseases that may cause vertigo.

In addition, audiometric data were also available from subjects using a clinical audiometer (AD229e, Interacoustics, Denmark), TDH39 headphones, and testing in a standard soundproof booth (< 30 dB A). Hearing status was indexed by the averaged pure tone hearing threshold (PTA) of the four frequencies (0.5/1/2/4 kHz) of the better ear.

Basic information for all subjects, including gender, age, and education, was collected before the test. For both patients and controls, individuals were excluded if they: (1) had hearing loss that affects daily communication; (2) had visual impairment; (3) had middle ear disease or long-term noise exposure; (4) had a history of psychiatric and/or neurological disorders such as anxiety and depression; (5) had years of education < 6 years; (6) had dementia disease, such as Alzheimer's Disease (AD).

### Visuospatial ability assessment

We used the Lenovo TB-J606F tablet with a resolution of 2,000 × 1,200 and a screen size of 11 inches to run the VCAS, which includes four modules: the “Weeding Test,” “Maze Test,” “Three-dimensional Driving (3D Driving),” and “Card Rotation Test.” Subjects were seated next to the experimenter with the tablet in front of them and completed tasks by touching the screen. Before any formal test, the subject is first directed through the “training mode” designed for each test to make sure that they have familiar with the processes. To avoid learning effects, the items in the training mode are different from those in the formal test. It takes approximately 40 min to complete all the tests. For patients, visuospatial assessment was performed before vestibular function tests. All test results will be stored in the Tencent Cloud storage bucket and displayed on the “Result Query” page allowing further analysis.

### Weeding test

**Figure 1:**
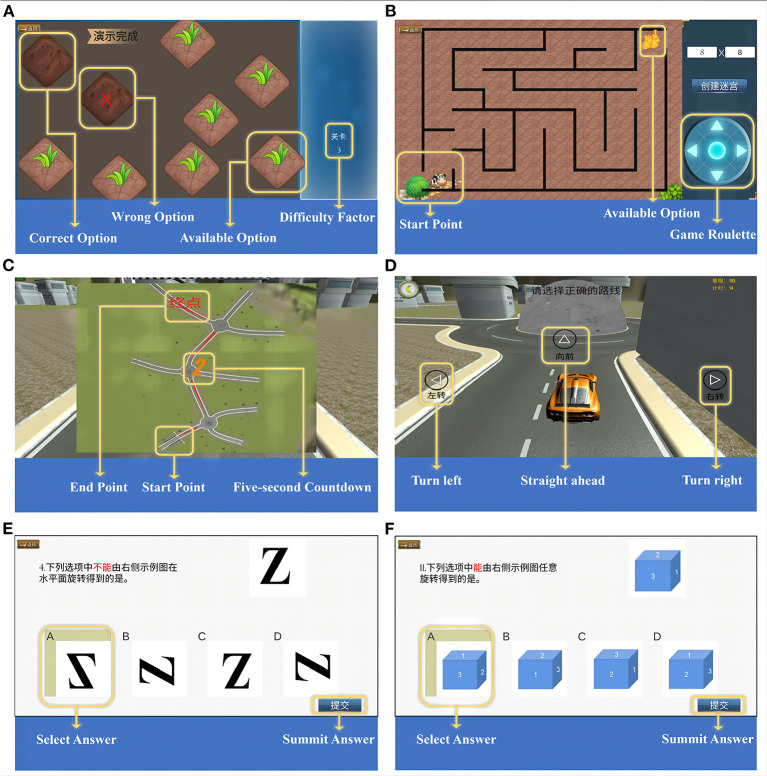
Visuospatial Cognition Assessment System (VCAS). **(A)** Diagram of weeding test. **(B)** Diagram of maze test, take map 8 × 8 as an example. **(C)** The map of three-dimensional driving (3D driving), and **(D)** The diagram of 3D driving, take one map as an example. **(E)** 2D-object of card rotation test, and **(F)** 3D-object of card rotation test.

We designed the “weeding test” inspired by the traditional Corsi Block Tapping paradigm and the work of Claessen et al. (31) and Lacroix et al. (32), which evaluates visuospatial attention and working memory processes. There are two sub-tests in the task: a forward and a backward condition. During the test, 9 squares symbolizing the “grass” were shown on the screen with a flashing time of 500 ms and an inter-block interval of 1 s. The relative block positions were the same as the traditional paradigm. Subjects have to memorize the sequence and click on the corresponding “grass” (i.e., weeding). In the forward condition, subjects need to reproduce the block sequence in the same serial order as indicated by the system, while in the backward condition, they repeat it in reverse order. Two trials per sequence length, ranging from two to nine blocks (for backwards, ranging from two to eight blocks). As long as the subject reproduce the block sequence completely correct in either of the two trials, one can enter the next sequence, and the game is over if the subject fails twice. The system automatically registered performance in terms of the span (forward/backward) which is used to evaluate the spatial working memory and the velocity (forward/backward), which is used to reflect the subject's click speed during the working memory process. The velocity was defined as the ratio of the total number of blocks clicked by the subject to the time spent in the test. The diagram is shown in Figure 1A.

### Maze test

Inspired by Lacroix et al. (32), we designed the “maze test” to assess spatial navigation and executive function. The map was randomly generated through math algorithm, which can be divided into numerous small squares (map “8 × 8,” i.e., this map can be divided into 64 small squares). The complexity of the map depends on the number of squares. Users can create different mazes for specific purposes by entering specific numbers in the input box at the top right of the screen and clicking the button “Create a maze.” According to the results of pre-experiment in a normal population, we included three maps of different difficulty levels (8 × 8, 10 × 10, and 12 × 12) here. At the subject level, the degree of difficulty is controlled by the number of corners. The calf in the lower left corner of the screen is the “starting point” and the straw in the upper right corner is the “destination.” Subjects have to move the calf to the “destination” by clicking the control panel in the lower right corner of the screen. There is only one correct route. All the subjects were instructed to get out of the maze as quickly as possible. The system automatically registered performance in terms of the time subjects took and the steps they moved for each maze. The diagram is shown in Figure 1B.

### Three-dimensional driving

Spatial memory and spatial navigation are often interdependent in real life. Inspired by Coutrot et al. (29), we designed “3D driving” to assess spatial memory and spatial navigation in a comprehensive manner. When the test begins, a two-dimensional map with all the intersections will be displayed in the center of the screen for 5 s. The subjects need to keep the map in mind and “drive” the car to the destination in a virtual three-dimensional scene according to the memorized route. Taking into account the limited operation ability of older people, we designed the autopilot mode to make the car move automatically and stop at each intersection. Only if the subjects click the buttons for direction selection (“turn left,” “forward,” and “turn right”) that appear on the screen correctly, the car will continue to drive. If the wrong selection was made, one will be prompted to re-select. To avoid learning effects, subjects have to complete three times (maps with different routes only) and the final results are averaged. The program automatically registered performance in terms of the thinking time and the number of errors at intersection. The diagram is shown in Figures 1C,D.

### Card rotation test

The “card rotation test” is inspired by traditional mental rotation paradigm and the work of Lacroix et al. (32) and Henn et al. (33). There are 8 questions in total. Each question takes a 2D or 3D figure as an example, and at the bottom of the screen are four figures with the same size and color but different rotation angles from the example. We used letters and numbers as 2D-object and blocks with numbers as 3D-object. Subjects were required to mentally switch to determine the option that was exactly the same or completely different from the example and click the corresponding button. There is only one correct answer. The same difficulty level included 2 questions. A correct answer is scored as 1 point, and an incorrect answer is scored as 0. The program automatically registered the performance in terms of the final score and the time spent on the subject. The diagram is shown in Figures 1E,F.

### Statistical analysis

All statistical analyses were performed with SPSS 25 (IBM; Armonk, NY, United States). The normality of distributions was evaluated using Shapiro-Wilk tests. Demographic data were analyzed with parametric analysis of variance (one-way ANOVA) for continuous data and chi-squared test for categorical data. We performed a multivariate ANOVA (for the weeding test and maze test) or multifactorial ANOVA (for the 3D driving test and card rotation test) followed by a *post-hoc* test (LSD) to compare the performance of different groups on each test of the VCAS. Each index was used as the dependent variable. Group, education, and gender were used as fixed factors, and only the factors with main effects were tested for interaction effects. For data do not meet the normal distribution, a ln logarithmic transformation is performed before ANOVA. To compare differences between subjects in the weeding test, two-way analyses of variance (ANOVAs) for repeated measures were performed on the span with 4^*^group (OVP, OC, MAC, YC) as a between-subject factor and 2^*^recall order (forward, backward) as a within-subject factor. The effect sizes are reported in terms of partial η^2^. An alpha level of 0.05 was used.

## Results

### Demographic and clinical characteristics

A total of 184 subjects participated in the study. Of these, 67 were clinical patients, and 117 were controls. According to the inclusion and exclusion criteria, 24 patients were excluded, including 15 with incomplete basic information, and 9 had normal vestibular test results (1 with a right ear perforation). Two controls with cataract were also excluded. Finally, 158 subjects (63.92% females) were included, including 43 patients (OVP; mean age: 66.14 years, SD: 4.5), 32 older controls (OC; mean age: 66.06 years, SD: 5.95), 38 middle-aged controls (MAC; mean age: 50.87 years, SD: 4.10), and 45 young controls (YC; mean age: 34.02 years, SD: 6.52). According to education, subjects were divided into primary education; secondary education, which includes middle school, high school, and technical secondary school; and higher education, which includes junior college, university, and postgraduate.

Age differences between the groups were statistically significant (*p* < 0.001). *Post-hoc* analyses indicated comparable ages between the OVP and the OC group. Gender differences were not statistically significant (*p* = 0.190). Education differences were statistically significant (*p* < 0.001). *Post-hoc* analyses showed no statistically significant difference between the OVP group and the OC group. Audiometric data was available in 116 of 158 subjects. The difference between the groups was statistically significant (*p* < 0.001). *Post-hoc* analyses showed that it was comparable between the OVP and the OC group as well as between the MAC and the YC group. Since the hearing thresholds of the OVP and OC groups were almost close to the normal range ( ≤ 25 dB HL), the hearing performance of the subjects was not further examined in this study. Demographic and hearing performance are presented in Table 1. Results of the vestibular function tests and hearing results of patients are shown in Supplementary Table S1.

**Table 1:** Demographic, and hearing performance of all groups.

	**OVP**	**OC**	**MAC**	**YC**	***P-*value**
	**(*N* = 43)**	**(*N* = 32)**	**(*N* = 38)**	**(*N* = 45)**	
**Age (mean, SD)**	66.14 (4.50)^a^	66.06 (5.95)^a^	50.87 (4.10)^b^	34.02 (6.52)^c^	**< 0.001**
**Sex (n, %)**					0.19
Male	17 (39.50)	16 (50.00)	11 (28.90)	13 (28.90)	
Female	26 (60.50)	16 (50.00)	27 (71.10)	32 (71.10)	
**Education (n, %)**					**< 0.001**
Primary education	2 (4.70)^a^	1 (3.10)^a^	7 (18.40)^a^	1 (2.20)^a^	
Secondary Education	29 (67.40)^a^	16 (50.00)^a^	15 (39.50)^a^	3 (6.70)^b^	
Higher Education	12 (27.90)^a^	15 (46.90)^a^	16 (42.10)^a^	41 (91.10)^b^	
**Hearing performance of the better ear (mean, SD in dB)**	28.9 (13.76)^a^	28.13 (14.97)^a^	16.2 (8.98)^b^	10.55 (7.48)^b^	**< 0.001**

OVP, Older vertigo patients; OC, Older controls; MAC, Middle-aged controls; YC, Young controls.

a−c Intergroup Comparison. Statistically significant results are bolded.

### Visuospatial cognitive outcomes between the OVP and OC group

**Figure 2:**
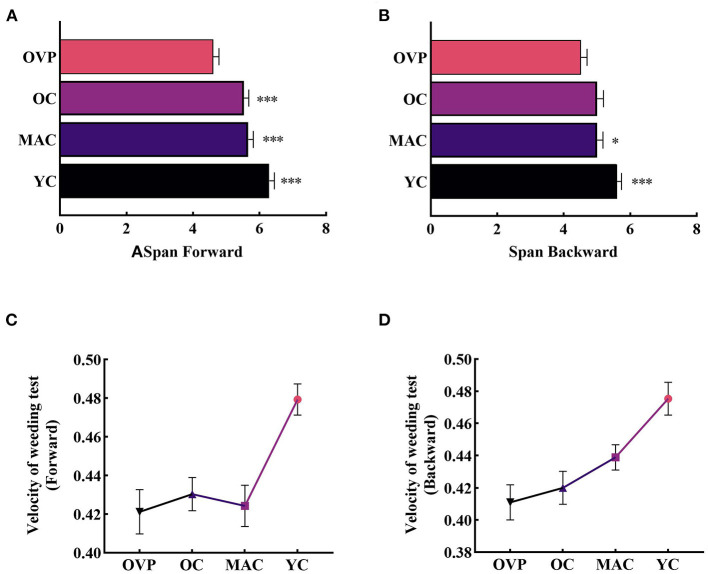
Span and Velocity of Weeding test between groups. **(A)** Span in the forward condition. **(B)** Span in the backward condition. **(C)** Velocity in the forward condition. OVP group only performed worse than YC group (*p* < 0.001). **(D)** Velocity in the backward condition. Older vertigo patients (OVP) performed worse than middle-aged controls (MAC) (*p* < 0.05) and young controls (YC) (*p* < 0.001). *Post-hoc* multiple comparisons between groups using OVP group as reference, significantly differences were indicated by the asterisk. OVP, Older vertigo patients; OC, Older controls; MAC, Middle-aged controls; YC, Young controls. *p < 0.05; ***p < 0.001. Error bars indicate the SEM.

For the weeding test, results displayed in Figure 2 and Table 2 show that only the factor group had a significant main effect on the span (F_*FW*_ = 7.062, *p* < 0.001, η^2^_*p*_ = 0.135; F_*BW*_ = 3.406, *p* = 0.020, η^2^_*p*_ = 0.070). *Post-hoc* analyses showed that the forward span was significantly shorter in the OVP group than in other groups (*p* < 0.001). For the backward span, there was no statistically significant difference between the OVP and OC groups (*p* = 0.057), but the OVP group performed significantly worse than the MAC group (*p* = 0.046) and the YC group (*p* < 0.001). Regarding the velocity, there was a main effect of group on backward condition (F_*FW*_ = 1.343, *p* = 0.263, η^2^_*p*_ = 0.029; F_*BW*_ = 2.709, *p* = 0.048, η^2^_*p*_ = 0.057), and a significant main effect of education on both forward and backward conditions (F_*FW*_ = 5.633, *p* = 0.004, η^2^_*p*_ =0.078; F_*BW*_ = 5.063, *p* = 0.008, η^2^_*p*_ =0.070). *Post-hoc* analyses showed the differences between the OVP and OC groups were nonsignificant in both conditions (FW, *p* = 0.654; BW, *p* = 0.527). Between the OVP and MAC groups, only the difference in the backward condition was significant (*p* = 0.043). Additionally, the OVP group performed significantly worse than the YC group in both conditions (*p* < 0.001). People with higher education levels had a faster click rate (FW, *p* = 0.004; BW, *p* = 0.008). The interaction between group and education was nonsignificant (F_*FW*_ = 0.817, *p* = 0.558, η^2^_*p*_ =0.055; F_*BW*_ = 1.008, *p* = 0.422, η^2^_*p*_ =0.057).

**Table 2:** Comparison of weeding test indexes between groups.

	**Span**		**Velocity**	
	**FW**	**BW**	**FW**	**BW**
	**Mean (SD)**	**Mean (SD)**	**Mean (SD)**	**Mean (SD)**
OVP	4.62 (1.15)	4.52 (1.15)	0.42 (0.07)	0.41 (0.07)
OC	5.53 (0.80)	5.00 (1.11)	0.43 (0.05)	0.42 (0.06)
MAC	5.66 (0.94)	5.00 (1.12)	0.42 (0.07)	0.44 (0.05)
YC	6.29 (1.04)	5.60 (0.91)	0.48 (0.05)	0.47 (0.07)
***P*** **-value**	**< 0.001**	**0.021**	0.263	**0.048**
				
Primary education	4.91 (1.45)	4.91 (1.14)	0.38 (0.08)	0.42 (0.07)
Secondary education	5.08 (1.00)	4.69 (1.10)	0.42 (0.06)	0.41 (0.06)
Higher education	5.95 (1.11)	5.32 (1.10)	0.46 (0.06)	0.46 (0.06)
***P*** **-value**	0.064	0.807	**0.004**	**0.008**

FW, forward; BW, backward; OVP, Older vertigo patients; OC, Older controls; MAC, Middle-aged controls; YC, Young controls. Statistically significant results are bolded.

**Figure 3:**
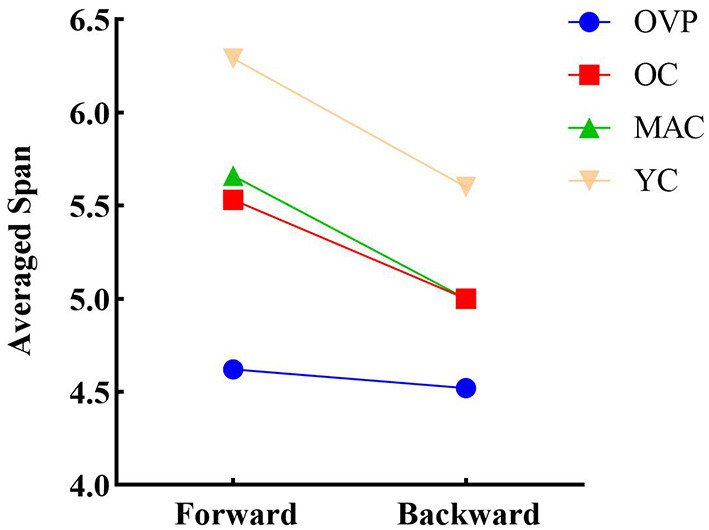
Differences in recall order of span for individual level. The forward span is significantly longer than the backward span (*p* < 0.001). Paired *t*-test revealed that the difference between recall order in OVP group was not statistically significant (*p* = 0.648). OVP, Older vertigo patients; OC, Older controls; MAC, Middle-aged controls; YC, Young controls.

Results of the two-factor repeated measures ANOVA revealed significant within-subjects effects (Figure 3). The forward span was significantly longer than the backward span (*F* = 21.159, *p* < 0.001, η^2^_*p*_ = 0.121). There was no interaction effect between group and recall order (*F* = 1.773, *p* = 0.155, η^2^_*p*_ = 0.034). That is, the difference between different recall orders did not change with the group. A paired *t*-test applied to the OVP group individually revealed no significant difference between the different recall orders (t = 0.461, *p* = 0.648).

**Figure 4:**
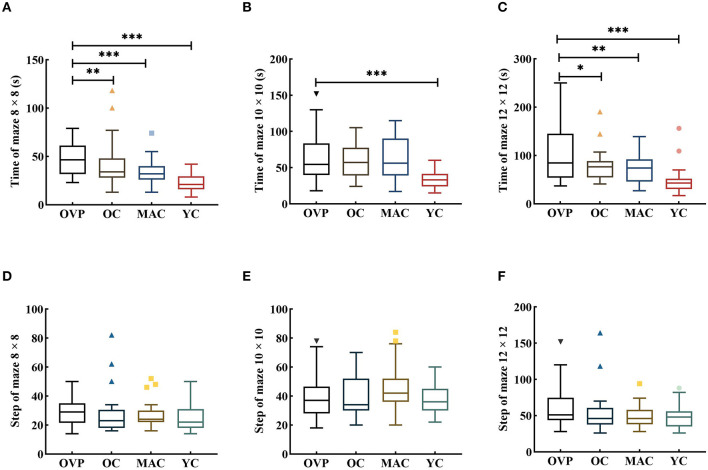
Time and Step of Maze test between groups. All the figures were Boxplot. The top and bottom lines of a column represented the maximum and minimum values of the data, respectively. The top and bottom lines of the box represented the third quartile and the first quartile, respectively, and the line in the middle of the box represents the median of the data. Colored symbols represented outliers. **(A–C)** Time of each group in different maps; **(D–F)** Step of each group in different maps. *Post-hoc* multiple comparisons between groups using OVP group as reference, significantly differences were indicated by the asterisk. OVP, Older vertigo patients; OC, Older controls; MAC, Middle-aged controls; YC, Young controls. *p < 0.05; **p < 0.01; ***p < 0.001.

Data in the maze test was transformed by the ln function before ANOVA. Results are shown in Figure 4 and Table 3. In all mazes, group had a significant effect on time [*F*_(8 × 8)_ = 5.995, *p* = 0.001, η^2^_*p*_ = 0.130; *F*_(10 × 10)_ = 7.666, *p* < 0.001, η^2^_*p*_ = 0.161; *F*_(12 × 12)_ = 9.690, *p* < 0.001, η^2^_*p*_ = 0.195]. *Post-hoc* analyses showed that the OVP group took significantly longer time than the OC group in different mazes except the maze 10 × 10 (8 × 8, *p* = 0.009; 10 × 10, *p* = 0.953; 12 × 12, *p* = 0.032). Gender had a main effect on the time for maze 10 × 10 and maze 12 × 12 [*F*_(10 × 10)_= 5.476, *p* = 0.021, η^2^_*p*_ = 0.044; *F*_(12 × 12)_ = 12.264, *p* = 0.001, η^2^_*p*_ = 0.093] expect the maze 8 × 8 [*F*_(8 × 8)_ = 2.662, *p* = 0.105, η^2^_*p*_ = 0.022]. There was no interaction effect between group and gender in all mazes [*F*_(8 × 8)_ = 0.004, *p* = 1.000, η^2^_*p*_ < 0.001; *F*_(10 × 10)_ = 0.104, *p* = 0.957, η^2^_*p*_ = 0.003; *F*_(12 × 12)_ = 0.212, *p* = 0.888, η^2^_*p*_ = 0.005]. Regarding the steps of the maze, no factors included in the analysis had main effect.

**Table 3:** Comparison of maze test indexes between groups.

	**Map 8** × **8**		**Map 10** × **10**		**Map 12** × **12**	
	**Time (s)**	**Step**	**Time (s)**	**Step**	**Time (s)**	**Step**
	**Median (IQR)**	**Median (IQR)**	**Median (IQR)**	**Median (IQR)**	**Median (IQR)**	**Median (IQR)**
OVP	46.50 (29.50)	29.00 (13.50)	54.50 (43.75)	37.00 (18.50)	84.50 (91.25)	51.00 (31.00)
OC	34.00 (23.00)	23.00 (12.50)	57.00 (38.50)	34.00 (22.00)	76.50 (34.00)	46.00 (23.00)
MAC	32.00 (14.00)	24.00 (8.00)	56.00 (51.00)	42.00 (16.00)	74.00 (46.00)	46.00 (20.00)
YC	21.00 (13.50)	22.00 (13.00)	33.00 (17.50)	36.00 (15.00)	43.00 (21.50)	48.00 (21.00)
***P*** **-value**	**0.001**	0.104	**< 0.001**	0.104	**< 0.001**	0.176
Male	29.00 (15.25)	23.00 (10.50)	40.50 (29.50)	37.00 (16.50)	58.00 (38.00)	48.00 (20.50)
Female	32.50 (25.50)	26.00 (12.50)	48.00 (38.50)	38.00 (18.00)	63.00(47.75)	49.00 (24.00)
***P-*** **value**	0.105	0.271	**0.021**	0.271	**0.001**	0.152

OVP, Older vertigo patients; OC, Older controls; MAC, Middle-aged controls; YC, Young controls. IQR, interquartile range. Statistically significant results are bolded.

**Figure 5:**
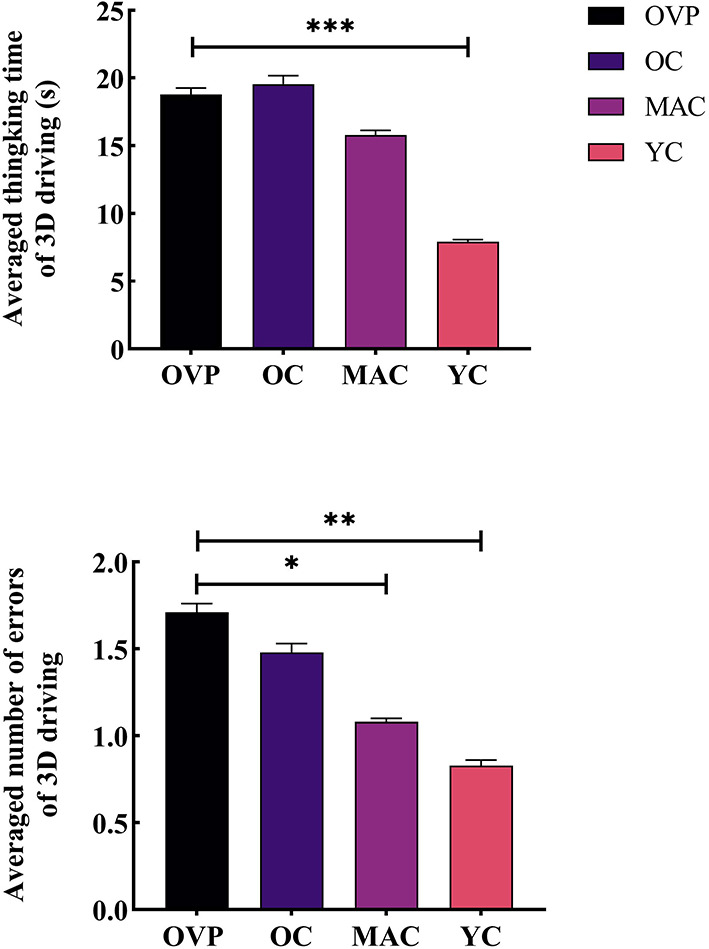
Time and errors of 3D driving test between groups. **(A)** Averaged thinking time of 3D driving. **(B)** Number of Errors of 3D driving. *Post-hoc* multiple comparisons between groups using OVP group as reference, significantly differences were indicated by the asterisk. OVP, Older vertigo patients; OC, Older controls; MAC, Middle-aged controls; YC, Young controls. *p < 0.05; **p < 0.01; ***p < 0.001. Error bars indicate the SEM.

Results of the 3D driving test are shown in Figure 5 and Table 4. There was a main effect of group and gender on thinking time (*F*_*group*_ = 5.177, *p* = 0.003, η^2^_*p*_ = 0.195; *F*_*gender*_= 5.472, p = 0.022, η^2^_*p*_ = 0.079). And there was no interaction effect between these two factors (*F* = 0.581, *p* = 0.630, η^2^_*p*_ = 0.027). *Post-hoc* analyses showed that the OVP group took longer time to think than the OC group, while the difference between the groups was nonsignificant (*p* = 0.774). Regarding the number of errors, there was only a main effect of group (*F* = 4.514, *p* = 0.006, η^2^_*p*_ = 0.151). *Post-hoc* analyses showed that the OVP group made a few more errors than the OC group, but the difference was nonsignificant (*p* = 0.399).

**Table 4:** Comparison of 3D driving test indexes between groups.

	**Map**	
	**Thinking time (s)**	**Errors**
	**Mean (SD)**	**Mean (SD)**
OVP	18.78 (7.36)	1.71 (1.02)
OC	19.56 (11.84)	1.48 (1.10)
MAC	15.81 (8.59)	1.08 (0.68)
YC	7.92 (3.45)	0.83 (0.79)
***P-*** **value**	**0.003**	**0.006**
Male	12.24 (7.49)	1.03 (0.87)
Female	16.59 (9.91)	1.34 (0.96)
***P-*** **value**	**0.022**	0.156

OVP, Older vertigo patients; OC, Older controls; MAC, Middle-aged controls; YC, Young controls. Statistically significant results are bolded.

**Figure 6:**
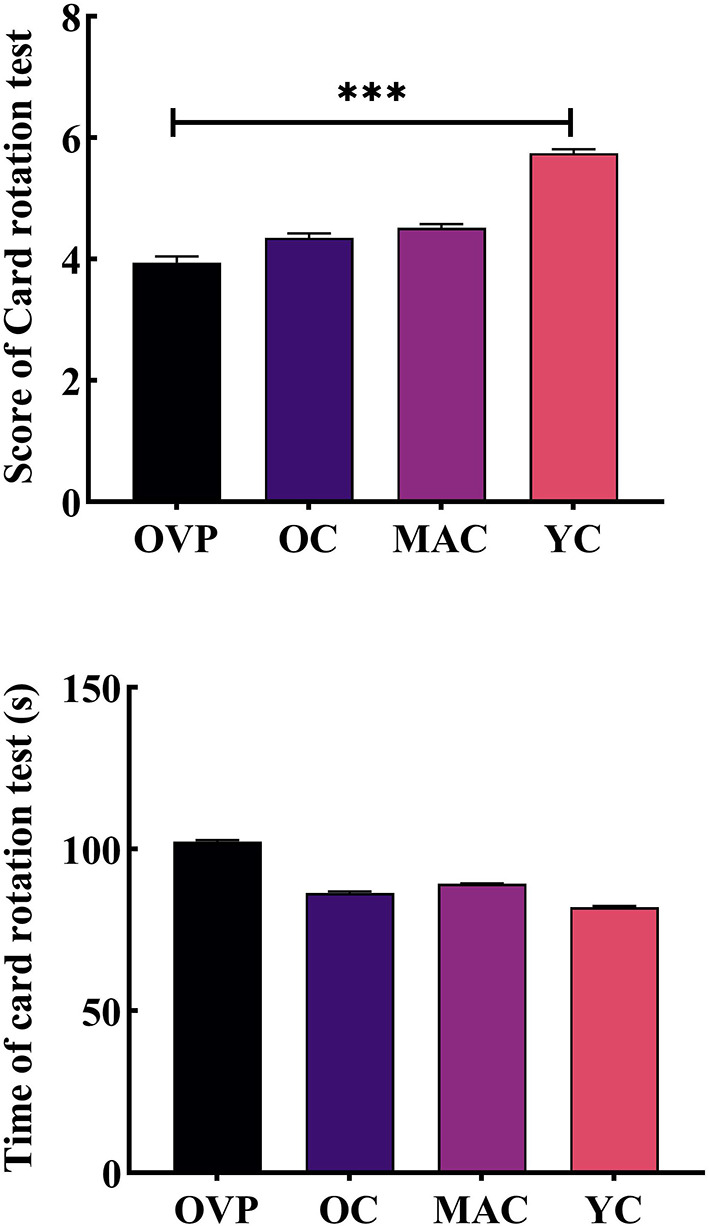
Score and time of Card rotation test between groups. **(A)** Score of card rotation test. **(B)** Time of card rotation test. *Post-hoc* multiple comparisons between groups using OVP group as reference, significantly differences were indicated by the asterisk. OVP, Older vertigo patients; OC, Older controls; MAC, Middle-aged controls; YC, Young controls. ***p < 0.001. Error bars indicate the SEM.

Results of the card rotation test are shown in Figure 6 and Table 5. There was no main effect of group on both score (*F* = 0.434, *p* = 0.729, η^2^_*p*_ = 0.021) and time (*F* = 0.157, *p* = 0.925, η^2^_*p*_ = 0.008). However, the OVP group indeed scored lower and took more time than the OC group, although the difference between the groups was nonsignificant (*p* = 0.400), while the difference in time between the groups was nearly statistically significant (*p* = 0.056). The results showed that only education had a main effect on the score (*F* = 3.190, *p* = 0.048, η^2^_*p*_ = 0.093). *Post-hoc* analyses showed that subjects with higher education scored a little higher than the other two groups.

**Table 5:** Comparison of card rotation test indexes between groups.

	**Card rotation test**	
	**Score**	**Score**
	**Mean (SD)**	**Mean (SD)**
OVP	3.94 (1.71)	102.29 (35.88)
OC	4.35 (1.27)	86.41 (34.47)
MAC	4.52 (1.39)	89.25 (28.07)
YC	5.74 (1.41)	82.11 (25.74)
***P-*** **value**	0.729	0.925
Primary education	4.25 (1.26)	80.00 (30.36)
Secondary education	4.00 (1.35)	96.35 (32.08)
Higher education	5.35 (1.51)	84.03 (29.59)
***P-*** **value**	**0.048**	0.807

OVP, Older vertigo patients; OC, Older controls; MAC, Middle-aged controls; YC, Young controls. Statistically significant results are bolded.

### Effects of aging on visuospatial cognitive outcomes in controls

For all the test metrics, we observed a stepwise increase in the performance between young controls, middle-aged controls, and older controls, although there were no significant differences between the OC group and the MAC group (Supplementary Figure S1). The YC group performed significantly better than the other groups separately in the span of the weeding test (FW: *p* = 0.001, *p* = 0.004; BW: *p* = 0.015, *p* = 0.011), the velocity of the weeding test (FW: *p* = 0.001, *p* < 0.001; BW: *p* < 0.001, *p* = 0.017), the time of the maze test (8 × 8: *p* < 0.001, *p* < 0.001; 10 × 10: *p* < 0.001, *p* < 0.001; 12 × 12: *p* < 0.001, *p* < 0.001) and the thinking time of the 3D driving (*p* < 0.001; *p* = 0.001). For the number of errors in the 3D driving, only the OC group had significantly more errors than the YC group (*p* = 0.014). In the card rotation test, although there was no main effect of group on both score and time (*F*_*score*_ = 0.434, *p* = 0.729, η^2^_*p*_ = 0.021; *F*_*time*_ = 0.157, *p* = 0.925, η^2^_*p*_ = 0.008), *post-hoc* analyses revealed that the YC group scored significantly higher than the OC group (*p* = 0.005) and the MAC group (*p* = 0.006), but the differences in time between all the control groups were nonsignificant.

## Discussion

In the present study, we assessed visuospatial ability through a portable computerized Visuospatial Cognition Assessment System (VCAS). We found that patients performed worse than the age-matched controls, but the differences of their performance on the 3D driving and card rotation tests were not statistically significant. Also, considering the importance of aging, we further divided the controls into different age groups and found that the older and middle-aged groups had comparable performance on all tasks, but both groups performed worse than the younger group on each task. Results suggest that: (1) Vestibular dysfunction is a risk factor for the decline in visuospatial ability, validating the link between the vestibular system and visuospatial ability; (2) Visuospatial ability declines with natural aging and may subsequently stabilize gradually; and (3) Assessing vestibular-related cognitive ability in a computerized way may be of great value in the future.

### Contribution of vestibular function on visuospatial cognitive outcomes

Spatial memory stores and manages information about the environment (e.g., location and relative position). We designed a computerized version of the Corsi block tapping task (CBT) (31) to evaluate visuospatial working memory (VSWM). Results found that the OVP group had a significantly shorter span than the age-matched controls in the forward condition. This is in line with previous work that short-term spatial memory was impaired in patients with chronic vestibular dysfunction during computerized CBT, with 4.11 ± 1.07 and 5.29 ± 0.77 for the forward span of older patients and controls, respectively (34). Unfortunately, they didn't talk about the backward condition. Our results showed that the OVP group indeed performed worse than the OC group in the backward condition, but the difference was not statistically significant, which may be due to the backward condition being more difficult and affecting the performance of controls. Regarding the differences between the forward and backward conditions, some studies argue that the backward condition, in which participants need to recall in the opposite order, increases the burden on cognition and is therefore more strenuous (27). However, similar results have been found between these two conditions (31), and one study even found that subjects performed better on the backward condition (35). In the present study, the forward span was significantly larger than the backward condition for controls, supporting the very first perspective. However, difference of the performance in the OVP group was nonsignificant, this may be due to the patients' poorer performance in the forward condition. There is still no consensus on whether these two conditions indeed tap into different cognitive processes. Future work is needed to further explore this issue.

For spatial navigation ability, results found that the OVP group took significantly longer time than the OC group. We did not observe a significant difference in steps between the two groups. It should be noted that during the test, we observed that more subjects in the OVP group took the wrong route and then retraced it to the correct one, which may lead to a significant increase in the time and steps. This can also be reflected on the larger interquartile range (IQR) of the OVP group. Our results confirm and extend prior reports regarding poorer performance in spatial orientation and spatial navigation in patients with vestibular dysfunction (9).

During the 3D driving test, although there were no statistically significant differences, the OVP group indeed made more errors than the OC group, suggesting that patients may have had some difficulties, such as poorer memory of routes or being more susceptible to getting confused. However, this did not seem to affect their ability to make the correct choice they thought. It is also to be noted that there were only three intersections in the map, which may have weakened the differences between groups.

As to mental rotation ability, we also did not find statistically significant differences between groups, although the OVP group still had a lower score and took longer time. This may be due to the highly diverse in terms of the type of vestibular diagnosis among patients, which was not sufficient to reflect the differences with controls in this task. Currently,the research on mental rotation ability is still controversial. Some studies have found that, BVP patients and patients with VN or BPPV may exhibit significant impairment in mental rotation task (10, 16). However, Nair et al. (36) found no significant differences in overall performance between BPPV patients and controls in a mental rotation task of 3D objects. More studies with large samples will be helpful for this issue in the future.

### Effect of aging on visuospatial ability in normal controls

With age, spatial orientation and navigational abilities may deteriorate (26). Collectively, in our study, the YC group had a better performance than other groups which is consistent with previous studies. However, the OC group and the MAC group had comparable performances during all the tests. We infer that visuospatial ability decreases with age and may gradually stabilize thereafter. It is intriguing that in the study by Coutrot et al. (29), there is an “inflection point” where performance on the navigation task improved for those over 75 years of age, and the authors explain that this could be a selective bias that these people may have extraordinary cognitive abilities.

The solid influence of aging on visuospatial abilities can be explained in terms of fluid intelligence (FUI), which refers to the ability to transfer and reason independently of prior experience and knowledge, and is more susceptible to aging than crystal intelligence, which relies on inherent experience accumulation (37). In addition, vestibular loss associated with normal aging may also mediate age-induced decline in cognition (38, 39). Therefore, it is not yet able to fully exclude the effects of age-related vestibular loss in this study. In the future, multicenter studies focusing on the effect of aging on visuospatial ability with larger sample size and finer age group delineation can help further clarify this issue.

### Computerized of test system

Previous studies have used a variety of instruments to assess visuospatial ability such as comprehensive scales or single-dimensional neuropsychological tests. Most of the tools are paper and pencil tests, which have some shortcomings: (1) It is difficult to control the experimental conditions accurately and is inconvenient for the management of test data. (2) The test conditions are mostly static, with little sense of dynamics. (3) Concentrate primarily on one dimension of visuospatial abilities. Furthermore, although some paradigms already have a computerized version, such as the computerized CBT (27, 31, 40), most of them are limited to the PC, which is inconvenient to carry for bedside evaluation. Therefore, we hope to develop an effective and convenient tablet-based test battery to provide a more dynamic and three-dimensional test condition and mainly focus on evaluating visuospatial ability, including spatial memory, spatial navigation, and mental rotation.

Our test system has specific advantages like other computerized task, such as precise control of the presentation interval and automatic recording of test metrics. However, we should be aware that there are still some potential issues. Firstly, it should be noted that there are possible differences between computerized versions and traditional test paradigms. For example, during the CBT, Popp et al. found a statistically significant difference between BVD patients and controls in the backward condition using a traditional tapping paradigm (41), which is different from the results we observed. One study has proposed that the hand movements of the experimenter in the traditional paradigm induce a motor-priming effect (31). The motor stimulus pre-presented will have either a positive or negative effect on the observer's motor processing, which indicates that the mental processes involved in the two modalities may differ and may limit the generalization of the results. Collectively, at least in the forward condition, there is an effect of vestibular dysfunction on spatial memory, and the use of the backward condition as a representative of spatial memory should clearly be reconsidered, especially in such computerized version. A larger number of subgroups using different modalities of CBT will be helpful to systematically investigate this issue.

Secondly, it has also been proposed that subjects do not rely on vestibular input from actual movement when completing such computerized task and only need to remain seated during the test, and attentional resources can be directed exclusively to the spatial memory task (42). Therefore, a purely static test setup may not be sufficient to capture the degree of impairment in real-life in patients with vestibular dysfunction. However, Kalová et al. (30) found that the results of a navigation test using a computerized version were similar to navigation performed in the real world, supporting the use of a computerized test modality to assess cognitive function.

In addition, the impact of subjects' familiarity and operation ability with electronic devices should be carefully considered when applying such computerized versions of the test, especially for older adults. To sum, although a computerized test system has been proven to have certain practical advantages, it is more sensible to be careful when quantitatively comparing visuospatial ability in virtual and real-world environments. Computerized versions of neuropsychological tasks should be carefully evaluated through a large-sample research design with normative data established for different age groups as well. And we believe that combine tablet-based test system and VR technology may improve our assessment tools more portable and effective in the future.

### Limitations

Of note, there are several limitations of the study. First, the cross-sectional design limits the causality of conclusions that can be drawn from it. Future work is needed to further explore such scenarios by developing longitudinal cohort studies; Secondly, the sample size of the subgroups in this study was small, which makes it difficult to further investigate the disease-specific effect within the OVP group and demonstrate a more refined trend in visuospatial abilities of controls; Finally, although the computerized version has certain advantages, it still lacks the input of dynamic vestibular information, which may not be sufficient to reflect the cognitive symptoms of patients.

## Conclusions

Vestibular cognition is a valuable area for clinical work. The current study presents evidence from a computerized Visuospatial Cognition Assessment System (VCAS) showing the close association between the vestibular system and visuospatial ability. Older adults with vestibular dysfunction have more difficulties during all tasks, especially spatial memory and spatial navigation. Visuospatial ability decreases with age and may gradually tend to be stable thereafter. Furthermore, computerizing the assessment of visuospatial ability is a valuable endeavor, but its effectiveness still should be treated with caution.

## Data availability statement

The raw data supporting the conclusions of this article will be made available by the authors, without undue reservation.

## Ethics statement

The studies involving human participants were reviewed and approved by the Ethical Committee of Peking University and the Peking University First Hospital (Approval No. 2021-390). The patients/participants provided their written informed consent to participate in this study.

## Author contributions

XZ designed and conceptualized the study, collected, analyzed, interpreted the data, designed, and drafted the manuscript. YL made a substantial contribution to the design and conception of this work and revised the manuscript critically for important intellectual content. YH participated in the collection and interpretation of the data and revising the manuscript. YX provided suggestions for experimental design, and revised the manuscript. XY participated in figure drawing. YZ, CW, and HY provided suggestions for experimental design. All authors contributed to the article and approved the submitted version.

## Funding

This research was supported by the Capital Clinical Special Application Research Project (Grant No. Z151100004015137).

## Conflict of interest

The authors declare that the research was conducted in the absence of any commercial or financial relationships that could be construed as a potential conflict of interest.

## Publisher's note

All claims expressed in this article are solely those of the authors and do not necessarily represent those of their affiliated organizations, or those of the publisher, the editors and the reviewers. Any product that may be evaluated in this article, or claim that may be made by its manufacturer, is not guaranteed or endorsed by the publisher.
