# Corrigendum: Vestibular dysfunction is an important contributor to the aging of visuospatial ability in older adults–Data from a computerized test system

**DOI:** 10.3389/fneur.2023.1166687

**Published:** 2023-03-30

**Authors:** Xuehao Zhang, Yan Huang, Yuqi Xia, Xiaotong Yang, Yanmei Zhang, Chaogang Wei, Hang Ying, Yuhe Liu

**Affiliations:** ^1^School of Medical Technology and Information Engineering, Zhejiang Chinese Medical University, Hangzhou, China; ^2^Department of Otolaryngology, Head and Neck Surgery, Peking University First Hospital, Beijing, China; ^3^Department of Otolaryngology, Head and Neck Surgery, Beijing Friendship Hospital, Capital Medical University, Beijing, China

**Keywords:** vestibular dysfunction, recurrent vertigo, aging, visuospatial ability, computerized test system

In the published article, there was an error in Materials and methods. Participants, paragraph 4. The stated inclusion criteria was not strict enough and previously stated “vertigo and characteristic positional nystagmus (torsional nystagmus in the Dix-Hallpike test, horizontal nystagmus in the Roll test) during the posturography, with the nystagmus lasting no more than 1 min.” This should have been “vertigo and characteristic positional nystagmus (torsional nystagmus in the Dix-Hallpike test, horizontal nystagmus in the Roll test) during the posturography.” The corrected paragraph appears below:

(2) Failed at least one of the following vestibular function tests:

- no recognizable P1 and N1 waves can be seen in either test ear at 100 dB SPL and/or bilateral asymmetry ratio (AR) of amplitude ≥1.6, measured by the c-VEMP.
- horizontal angular VOR gain < 0.8 (< 0.7 for vertical direction) with saccade wave, measured by the v-HIT.
- vertigo and characteristic positional nystagmus (torsional nystagmus in the Dix-Hallpike test, horizontal nystagmus in the Roll test) during the posturography.
- reduced caloric response (sum of bithermal, 24 and 50°C maximum peak slow phase velocity (SPV) on each side < 12°/s), and/or unilateral weakness (UW) ≥25%.

In the published article, the reference “Guidetti G, Guidetti R, Manfredi M, Manfredi M. Vestibular pathology and spatial working memory. *Acta Otorhinolaryngol Ital*. (2020) 40:72–8. doi: 10.14639/0392-100X-2189,” was not cited in the article. The citation has now been inserted as reference (34) in Discussion, Contribution of vestibular function on visuospatial cognitive outcomes, paragraph 1 and should read:

This is in line with previous work that short-term spatial memory was impaired in patients with chronic vestibular dysfunction during computerized CBT, with 4.11 ± 1.07 and 5.29 ± 0.77 for the forward span of older patients and controls, respectively (34).

In the published article, there was also an error in Supplementary Table 1 as published. In the table “Hearing performance of the better ear” should have been “Hearing performance of the better ear (*n*, %)”. Supplementary Table 1 has been updated in the Supplementary material of the published article.

The authors apologize for these errors and state that this does not change the scientific conclusions of the article in any way. The original article has been updated.

