# Supplementary material for: Vestibular dysfunction is an important contributor to the aging of visuospatial ability in older adults–Data from a computerized test system
Source: Front Neurol. 2022 Nov 17;13:1049806. doi: 10.3389/fneur.2022.1049806 (PMC9714458; doi:10.3389/fneur.2022.1049806)
Supplement: Supplementary file 1 [file Data_Sheet_1.docx]

Supplementary Material


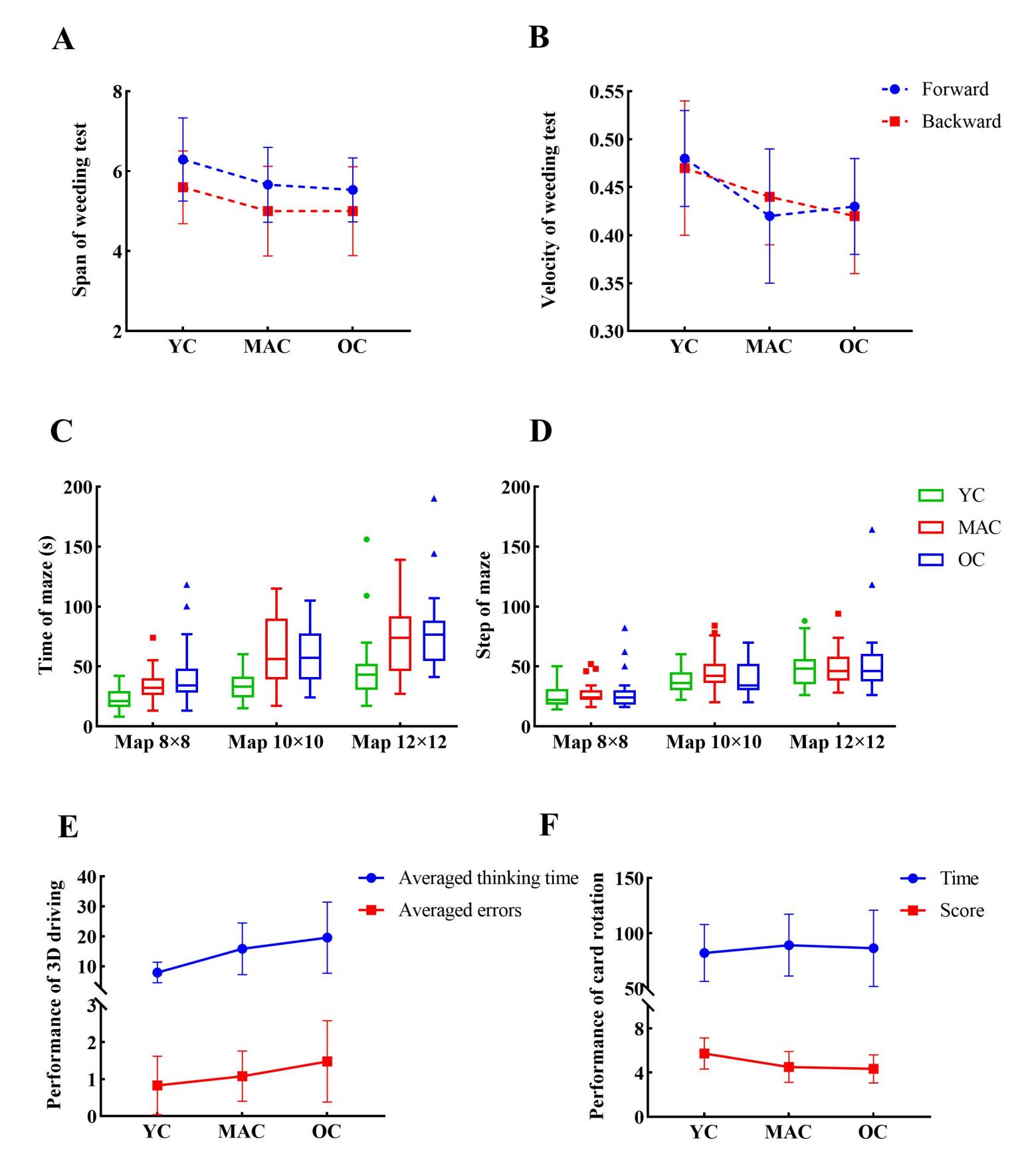
Supplementary Figures

## Supplementary Figure 1. Visuospatial cognitive outcomes in normal controls. (A) Span in both forward and backward conditions rose as age increased. (B) Velocity in both forward and backward conditions rose as age increased. (C) Time of maze test rose as age increased. Younger controls (YC) performed better than middle-aged controls (MAC) and older controls (OC). (D) The performance on the steps of the maze test were comparable between all the age groups. (E) Younger controls (YC) took less thinking time than the middle-aged controls (MAC) and older controls (OC), while the performance on the averaged errors of 3D driving were comparable. (F) The performance of card rotation test was comparable between all the age groups. YC, Young controls; MAC, Middle-aged controls; OC, Older controls. Error bars indicate the SD.

## Supplementary Tables

| **Supplementary TABLE** **1** **\|** Results of the vestibular function tests and hearing results of clinical patients. | |
| --- | --- |
| **c-VEMP function category (n, %)** | ***N* = 17** |
| Present | 8 (47.06)^a^ |
| Unilaterally absent | 3 (17.65) |
| Bilaterally absent | 6 (35.29) |
| **VOR gain category (n, %)** | ***N* = 18** |
| Normal | 7 (38.89) |
| Unilaterally abnormal | 7 (38.89) |
| Bilaterally abnormal | 4 (22.23) |
| **Posturography (n, %)** | ***N* = 22** |
| Negative | 15 (68.18) |
| Positive | 7 (31.82) |
| **Caloric irrigation (n, %)** | ***N* = 23** |
| Normal | 14 (60.87) |
| Unilaterally abnormal | 9 (39.13) |
| Bilaterally abnormal | 0 |
| **Hearing performance of the better ear (n, %)** | ***N* = 40** |
| Normal | 21 (52.50)^b^ |
| Mild hearing loss | 13 (30.50) |
| Moderate hearing loss | 5 (12.50) |
| Severe hearing loss | 1 (2.50) |
| Profound hearing loss | 0 |

Patients were recruited from the Neurotology Clinic, aged 60 years and older. ^a^One patient had bilateral amplitude asymmetry with bilateral asymmetry ratio (AR) ≥ 1.6. ^b^Two patients had unilateral deafness. c-VEMP, cervical vestibular-evoked myogenic potential; VOR, vestibulo-ocular reflex.
